# Differential regulation of the androgen receptor by protein phosphatase regulatory subunits

**DOI:** 10.18632/oncotarget.22883

**Published:** 2017-12-04

**Authors:** James Grey, Dominic Jones, Laura Wilson, Sirintra Nakjang, Jake Clayton, Richard Temperley, Emma Clark, Luke Gaughan, Craig Robson

**Affiliations:** ^1^ Northern Institute for Cancer Research, The Medical School, Newcastle University, Framlington Place, Newcastle upon Tyne, NE2 4HH, UK

**Keywords:** androgen receptor, prostate cancer, castrate resistant prostate cancer, phosphatase, myosin phosphatase

## Abstract

The Androgen Receptor (AR) is a key molecule in the development, maintenance and progression of prostate cancer (PC). However, the relationship between the AR and co-regulatory proteins that facilitate AR activity in castrate resistant settings remain understudied. Here we show that protein phosphatase 1 regulatory subunits, identified from a phosphatase RNAi screen, direct PP1 catalytic subunits to a varied yet significant response in AR function. As such, we have characterised the PP1β holoenzyme, myosin phosphatase (MLCP), as a novel ligand independent regulator of the AR. Sustained MLCP activity through down-regulation of the MLCP inhibitory subunit, PPP1R14C, results in impaired AR nuclear translocation, protein stability and transcriptional activity in distinct models of PC progression, culminating in restoration of a non-malignant prostate genotype. Phenotypically, a marked reduction in cell proliferation and migration, characterised by G1 cell cycle arrest is observed, confirming PP1 holoenzyme disruption as a novel treatment approach in PC.

## INTRODUCTION

Prostate cancer (PC) is the most common non-cutaneous cancer in males in western countries and is fast emerging as a significant health risk in the developing world, accounting for the second highest rate of cancer related deaths in men worldwide (www.cancerresearch.co.uk). Androgen deprivation therapy (ADT), aimed at disrupting the androgen signalling axis, is initially successful [1], with the vast majority of patients demonstrating tumour regression. Unfortunately, up to 95% of patients relapse within a median time of 2 years [2], with disease progressing to an androgen-independent state termed castrate resistant PC (CRPC), characterised by increased incidence of metastasis and ultimately death. However, it has become increasingly evident that the androgen receptor (AR) is still capable of driving disease progression through re-activation of the androgen signalling axis, and as such remains a viable therapeutic target [3]. Numerous routes to resistance have been described thus far including *AR* gene amplification [4], the acquisition of *AR* somatic mutations and emergence of receptor splice variants [5].

AR activity is regulated at numerous levels by the activity of co-activator and co-repressor proteins [6] and uncontrolled activity of a number of these co-regulators has been postulated to enable progression to CRPC [7–9]. To date, the impact of protein kinases on AR function has been extensively studied (reviewed in [10]), with modulation of AR activity via the inhibition of upstream kinases including Aurora A [11], CDK1 [12] and MAPK [13], representing proven examples that AR co-activator inhibition may represent a viable therapeutic approach in PC and CRPC. In contrast, the opposing activity of dephosphorylation, performed by phosphatase enzymes, is markedly understudied, with only a limited number of phosphatases described as being capable of modulating AR activity. Recent literature has implicated protein phosphatase 1α (PP1α) as an AR co-activator capable of promoting AR stability, nuclear localization and transcriptional potency, but to date has displayed little therapeutic potential due to the toxic nature of current inhibitors targeting the PP1 catalytic subunits [14–16]. However, PP1 catalytic subunits do not exist as a monomeric subunits *in-vivo,* but rather, associate with a wide range of mutually exclusive regulatory subunits to form PP1 holoenzymes; each with distinct substrate specificities, subcellular localizations, and catalytic activities [17]. This provides the PP1 catalytic subunit with a dynamic means of counteracting the highly specific and sensitive action of hundreds of kinases. Myosin Phosphatase (MLCP) is one such PP1 holoenzyme, comprising the PP1β catalytic subunit, PPP1R12A regulatory subunit, and a small 20 kDa protein of unknown function to form a heterotrimeric complex [18, 19]. PPP1R12A acts as a regulatory subunit to PP1β by binding to a highly specific subset of PP1 substrates, and bringing the substrate and catalytic subunit into contact. As its name would suggest, MLCP effectively dephosphorylates phosphorylated myosin regulatory light chain (MLC), and as such plays a pivotal role in actomyosin contractility. However, a broad range of signalling molecules have subsequently been identified as substrates for MLCP, including RB1, NF2, PLK1, HDAC7 and tau [20–25]. Interestingly, MLCP activity can be potently inhibited following association of the MLCP complex with one of the PPP1R14 family of MLCP inhibitory proteins, that upon phosphorylation behave as pseudosubstrates for this specific PP1 holoenzyme [26–28].

Through the implementation of a human phosphatome RNAi screen we demonstrate for the first time that the PP1b catalytic subunit is repressive towards AR function, but more specifically, that the impact of PP1 activity on AR function is entirely dependent upon the association of the catalytic subunit with its respective regulatory subunits. As such, we characterise the PP1β holoenzyme MLCP as a novel dynamic regulator of the AR, and demonstrate that through downregulation of the endogenous MLCP regulatory inhibitory subunit, PPP1R14C [28], ligand induced AR nuclear translocation, stability, and ultimately AR transcriptional activity is significantly reduced in distinct PC cell line models representing both androgen sensitivity and castration resistance. Phenotypically we are able to show that depletion of PPP1R14C reduces PC cell growth and migration characterised by G1 cell cycle arrest. Conversely, depletion of PPP1R12A results in increased AR mRNA expression, protein expression and AR transcriptional activity in both the presence and absence of androgen, confirming MLCP as a novel dynamic ligand independent regulator of the AR. With this in mind, we propose that the disruption of specific PP1 holoenzymes provides a distinct route of PP1 inhibition, and as such, a viable approach in the treatment of PC and CRPC.

## RESULTS

### Human phosphatome RNAi screen reveals distinct roles for protein phosphatase 1 regulatory subunits in the regulation of androgen receptor activity

**Figure 1:**
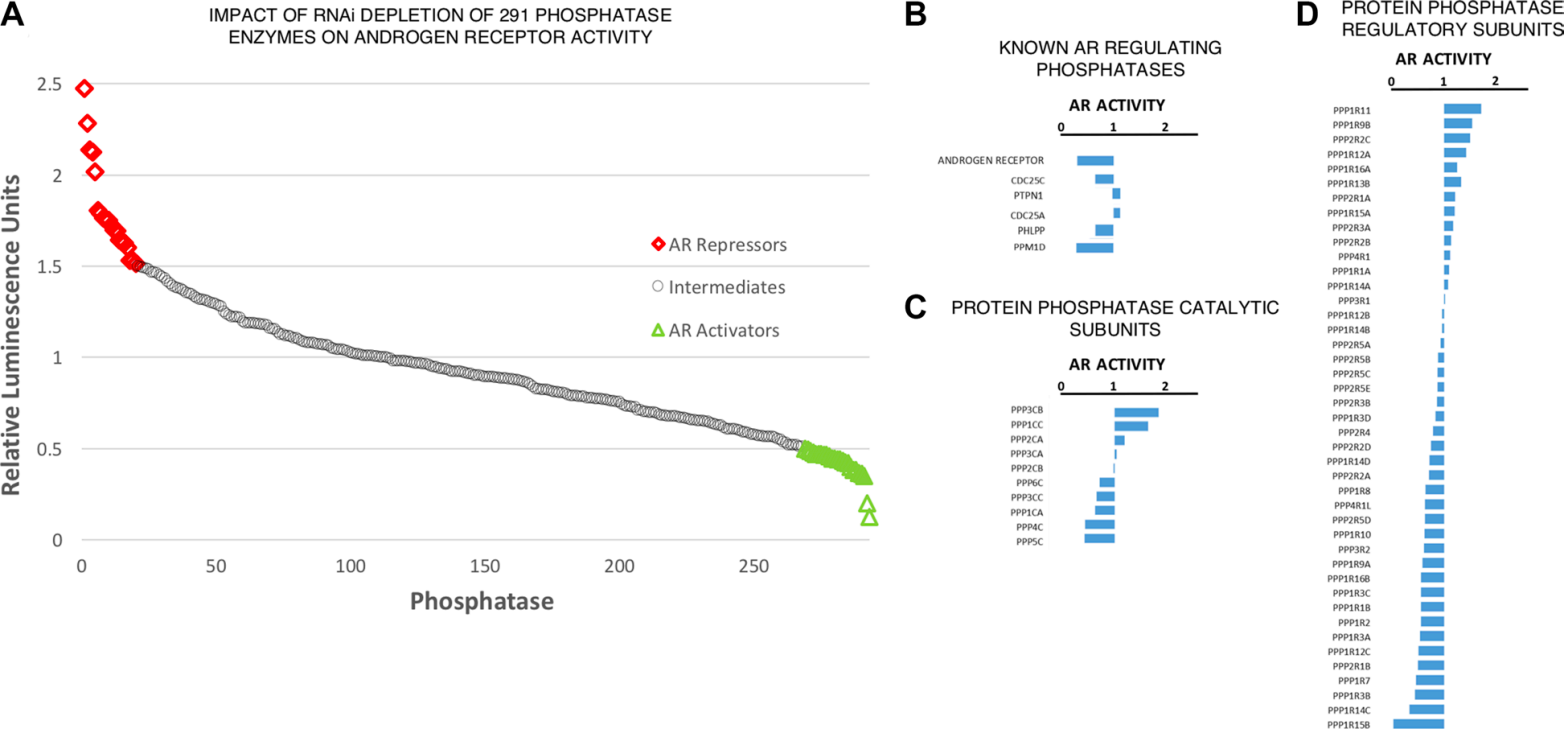
Phosphatase regulatory subunits differentially impact upon AR activity (**A**) Impact of phosphatase depletion on AR transcriptional activity. AR activity represents mean luciferase activity (Fold-Change, *n* = 3). Full dataset (281 targets) found in Supplementary Table 1. (**B**) Impact of AR and previously identified AR modulating phosphatase depletion on AR transcriptional activity. (**C**) Impact of protein phosphatase catalytic subunit depletion on AR transcriptional activity. (**D**) Impact of protein phosphatase regulatory subunit depletion on AR transcriptional activity.

Post-translational modifications of the AR have been widely studied to investigate the functional interplay between the AR and co-activators or co-repressors for therapeutic benefit (reviewed [29]). However, we identified a significant knowledge gap in the role of phosphatase enzymes in AR regulation. In order to address this we performed an RNAi screen individually targeting 291 phosphatase enzymes and phosphatase associated proteins, and assessed the impact on AR transcriptional activity using a derivative of the androgen-responsive cell line LNCaP that stably over-expresses luciferase under the control of the *PSA* gene promoter (termed LNCaP-PSALuc). Figure 1A depicts the impact of target knockdown (mean, *n* = 3) on luciferase activity, used as a surrogate for AR activity (detailed luciferase activity for all 291 targets can be found in Supplementary Table 1). RNAi depletion of the AR and previously identified AR regulators Figure 1B, including PP1a and PP2A Figure 1C, yielded the expected outcome in terms of AR activity, thus validating the results of the screen with known research [15, 16, 30, 31]. Interestingly, depletion of the PP1b catalytic subunit significantly reduced AR transcriptional activity, suggesting a contrasting role in AR regulation to the closely related PP1a subunit. However, it became clear that RNAi knockdown of PP1 regulatory subunits resulted in distinct, yet highly significant, modulation of AR activity, revealing a more complex role for PP1 holoenzymes in the regulation of AR activity than previously described (Figure 1D). Furthermore, PP1 regulatory subunits accounted for 17.5% of the most significant AR stimulatory factors (*n* = 13/75). As previous research has implemented RNAi depletion or small molecule inhibition of the PP1 catalytic subunits in AR activity studies, eliminating the full repertoire of PP1 holoenzymes, we sought to characterise the individual roles of regulatory subunits on AR function. This approach would enable our research to elucidate the specific PP1 holoenzymes responsible for the observed impacts on AR activity.

### Components of the PP1β holoenzyme myosin phosphatase are dysregulated in human prostate cancer

**Figure 2:**
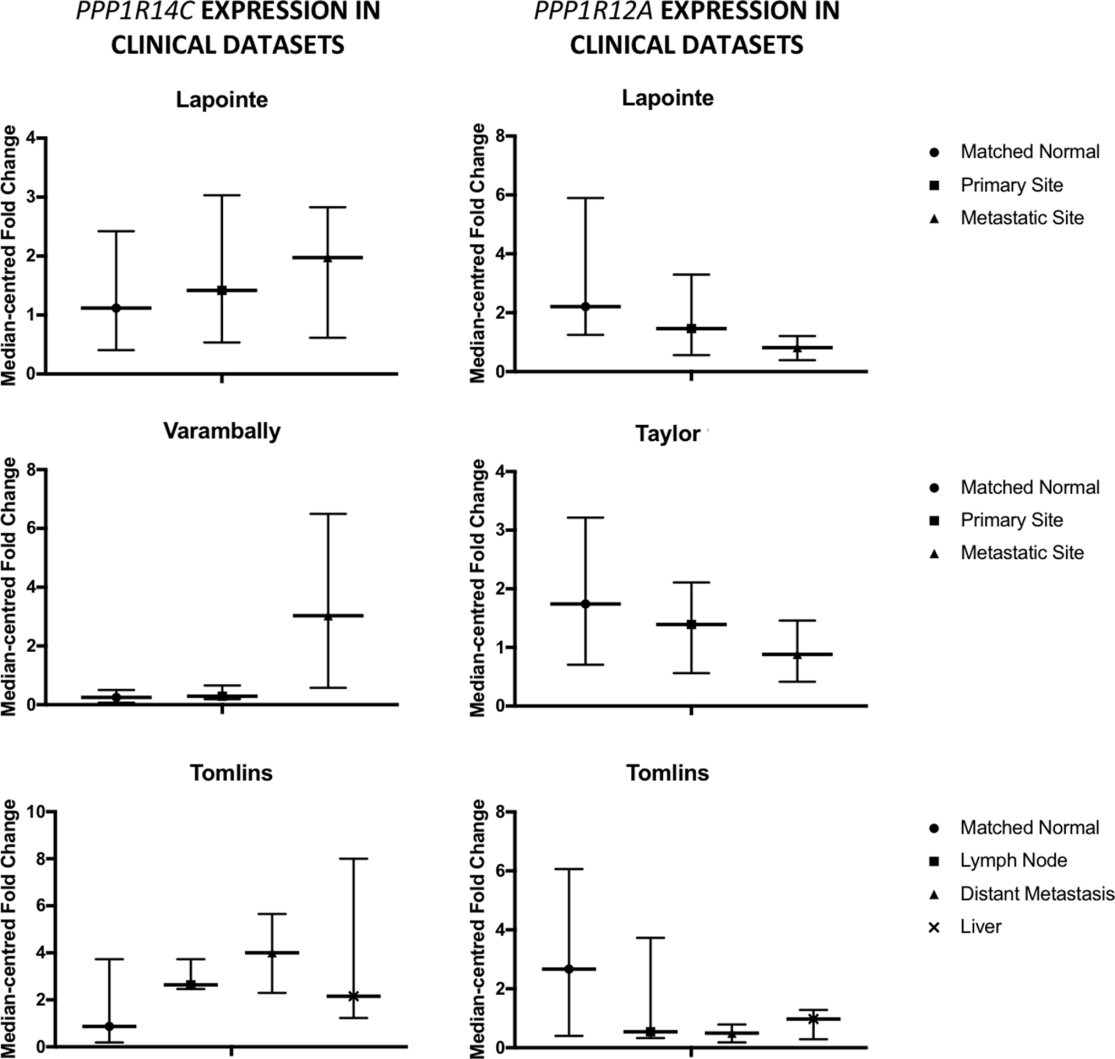
Myosin phosphatase components are dysregulated in prostate cancer Expression of PPP1R14C and PPP1R12A is evaluated in 4 publicly available prostate cancer datasets.

We evaluated the expression of PP1 regulatory subunits in publicly available prostate cancer datasets. Whilst evidence existed for inter-patient variation in the expression of the PP1 regulatory subunit cohort, it became apparent that two components of the PP1β holoenzyme MLCP were consistently dysregulated in multiple datasets analysed (Figure 2 [32–35]). PPP1R12A, the substrate specifying subunit for MLCP, was identified as being down-regulated in PC vs matched normal prostate, but more specifically, down-regulated at sites of metastasis vs primary tumours and matched normal prostate. Conversely, PPP1R14C, an inhibitory subunit for MLCP, was found to be up-regulated in PC vs matched normal prostate, particularly at metastatic sites. Interestingly, these expression trends occurred both independently and combined. As PPP1R14C is a negative regulator of MLCP activity at the post-translational level, this data would suggest a net loss of MLCP activity exists throughout the emergence and progression of PC to metastatic PC. Here, PPP1R14C and PPP1R12A were identified as novel AR activators and repressors in our phosphatase RNAi screen respectively. As previously mentioned, regulation of the PP1 catalytic subunits relies upon the dynamic association with their respective regulatory subunits, and as such exist as up to 200 distinct holoenzymes [36]. In this instance, PPP1R12A provides substrate specificity to the PP1b catalytic subunit, forming the holoenzyme MLCP. Indeed, two recent independent research articles have identified miR-30d as a prognostic marker in PC, conferring enhanced proliferation, invasion and significantly shorter time to biochemical recurrence [37, 38]. Interestingly, PPP1R12A has been identified as the direct target of miR-30D. Similarly, a novel oncogenic pathway has recently been described in invasive lobular breast cancer, whereby truncating mutations in PPP1R12A promote the malignant transformation of E-cadherin deficient mammary epithelial cells by impairing the ability of MLCP to effectively dephosphorylate its substrates [39]. In addition to genomic and transcriptional events, the activity of MLCP can be modulated post-translationally upon association with the highly specific MLCP inhibitory PPP1R14 family of subunits. PPP1R14A-D family members are not intrinsically inhibitory towards MLCP, but upon phosphorylation by a number of kinases, become potent inhibitory pseudosubstrates specific to MLCP following a 600–1000 fold increase in their affinity for the MLCP complex. Indeed, PPP1R14A is overexpressed in a number of human malignancies, and it has been demonstrated that increased expression of PPP1R14A is sufficient to induce tumorigenic transformation in multiple cell lines, characterised by a reduction in MLCP activity [22]. Similarly, PPP1R14B has been reported to be overexpressed in ovarian clear cell carcinoma [40], as well as serving as an unfavourable prognostic marker in liver and pancreatic cancers. Furthermore, we also observe significant overexpression of PPP1R14B in human prostate cancer following interrogation of publicly available datasets.

### Depletion of PPP1R14C reduces AR transcriptional activity in distinct cell line models of prostate cancer

**Figure 3:**
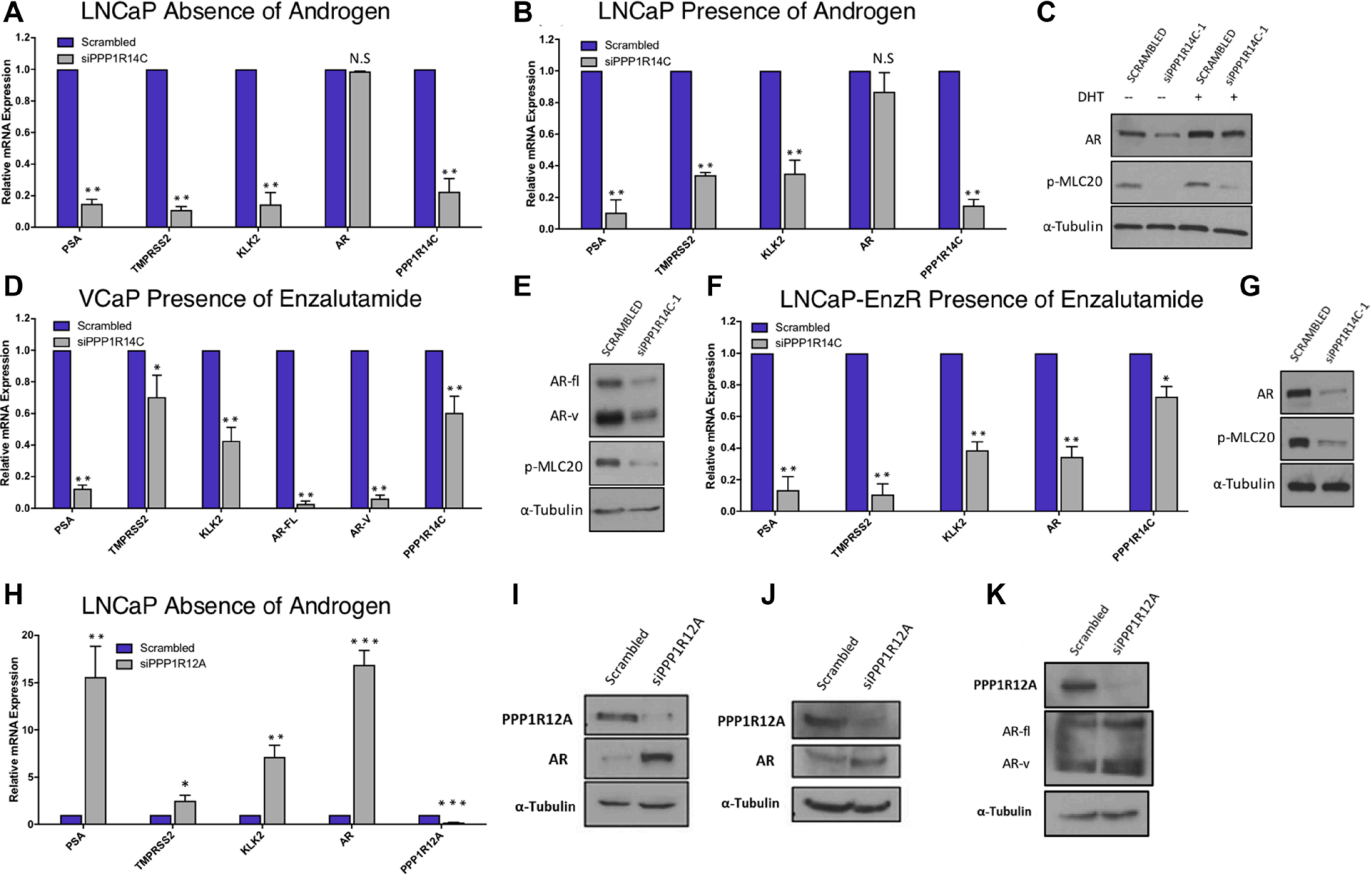
PPP1R14C RNAi depletion reduces AR transcriptional activity in distinct cell line models of prostate cancer (**A–B**) Impact of PPP1R14C RNAi depletion on *PSA, TMPRSS2, KLK2* and *AR* gene expression in LNCaP cells cultured in steroid depleted media ± 10 nM DHT. (**C**) Western blot analysis of AR and pMLC20 following PPP1R14C RNAi depletion in LNCaP cells in the presence and absence of androgen. (**D**) Impact of PPP1R14C RNAi depletion on *PSA, TMPRSS2, KLK2,* full-length *AR* and *AR-V* gene expression in VCaP cells cultured in full media supplemented with 10 µM enzalutamide. (**E**) Western blot analysis of AR and pMLC20 following PPP1R14C RNAi depletion in VCaP cells in the presence of 10 µM enzalutamide. (**F**) Impact of PPP1R14C RNAi depletion on *PSA, TMPRSS2, KLK2* and *AR* gene expression in LNCaP-EnzR cells cultured in full media supplemented with 10 µM enzalutamide. (**G**) Western blot analysis of AR and pMLC20 following PPP1R14C RNAi depletion in LNCaP-EnzR cells in the presence of 10µM enzalutamide. (**H**) Impact of PPP1R12A RNAi depletion in LNCaP cells in the absence of androgen. (**I–J**) Western blot analysis of PPP1R12A and AR following PPP1R12A RNAi depletion in LNCaP cells in the presence and absence of androgen. (**K**) Wetern blot analysis of PPP1R12A and AR following PPP1R12A RNAi depletion in VCaP cells in full media. Data represents 3 independent experiments ± SEM. *P*- values were determined by unpaired student *t* test (^**^, ^*^ denote *P*-values of < 0.001 and < 0.01 respectively).

Data obtained from the phosphatase RNAi screen in LNCaP-PSALuc cells suggested that PPP1R14C depletion reduced AR transcriptional activity by 60%. To confirm this, PPP1R14C was knocked down in several additional PC cell line models representing different levels of androgen sensitivity. As knockdown of PPP1R14C with 3 independent RNAi oligos resulted in significant repression of both *PPP1R14C* and AR target gene mRNA expression in LNCaP cells (Supplementary Figure 1), siPPP1R14C-1 was taken forward for future assays. Furthermore, due to a lack of commercial antibodies available for PPP1R14C, enhanced activity of MLCP towards its substrate phosphorylated myosin has been used as a surrogate for PPP1R14C depletion at the protein level. PPP1R14C depletion in parental LNCaP cells significantly reduced AR transcriptional activity as indicated by down-regulation of receptor target genes *PSA, TMPRSS2* and *KLK2* (Figures 3A and 3B). Furthermore, it is possible to demonstrate that PPP1R14C RNAi knockdown results in reduced AR protein expression when compared to the scrambled control, as depicted in Figure 3C. Critically, AR protein expression and activity was compromised by PPP1R14C depletion in both the presence and absence of dihydrotestosterone (DHT), suggesting that MLCP modulates AR function in a ligand independent manner.

Next we investigated the impact of PPP1R14C depletion in the VCaP CRPC cell line. Similarly, we were able to demonstrate that depletion of PPP1R14C is capable of significantly repressing AR transcriptional activity in both the presence and absence of androgen as depicted in Supplementary Figure 2. The emergence of AR splice variants is a clinically relevant treatment resistance mechanism [41], and in order to assess the impact of PPP1R14C on AR-V activity, VCaP cells underwent PPP1R14C manipulation in the presence of 10mM enzalutamide which specifically targets full length, ligand binding domain-containing AR. Crucially, we were able to show that AR activity was diminished following RNAi depletion of PPP1R14C (Figure 3D), consistent with the notion that MLCP is a ligand independent regulator of the AR, and that MLCP activity is also repressive to constitutively active AR splice variants. The impact of PPP1R14C depletion on AR transcriptional activity in VCaPs correlates with a reduction in the steady state protein levels of full length AR and splice variant AR, as observed in Figure 3E. Finally, to further confirm that MLCP may be capable of repressing aberrant AR activity, PPP1R14C RNAi depletion was performed in an enzalutamide resistant LNCaP cell line (LNCaP-EnzR), generated in house, that maintains an active, and responsive, androgen signalling axis in the presence of this particular anti-androgen. Crucially, significant repression of AR target gene expression was observed upon knockdown of PPP1R14C in the presence of 10 mM enzalutamide supporting the concept that the liganded and unliganded full-length AR, as well as AR-Vs, are regulated by the PPP1R14C-MLCP axis, and provides evidence that PPP1R14C modulation may be a viable therapeutic target for treatment of advanced CRPC (Figure 3F and 3G).

To investigate the impact of diminished MLCP activity on AR function, PPP1R12A was depleted by RNAi. Crucially, in LNCaP cells we demonstrate a significant increase in AR mRNA and protein expression following PPP1R12A depletion in both the presence and absence of androgen, culminating with significantly increased AR transcriptional activity (Figure 3H, 3I, 3J and Supplementary Figure 3). Indeed, what is most surprising is that the increases in AR expression and activity are most pronounced in the absence of androgen, suggesting MLCP inhibition may serve as a route to castration resistance. Furthermore, we are able to demonstrate that PPP1R12A RNAi depletion in VCaP cells results in increased protein expression of both full-length and splice variant AR (Figure 3K).

### PPP1R14C depletion prevents ligand induced AR nuclear translocation and promotes AKT mediated proteasomal degradation

**Figure 4:**
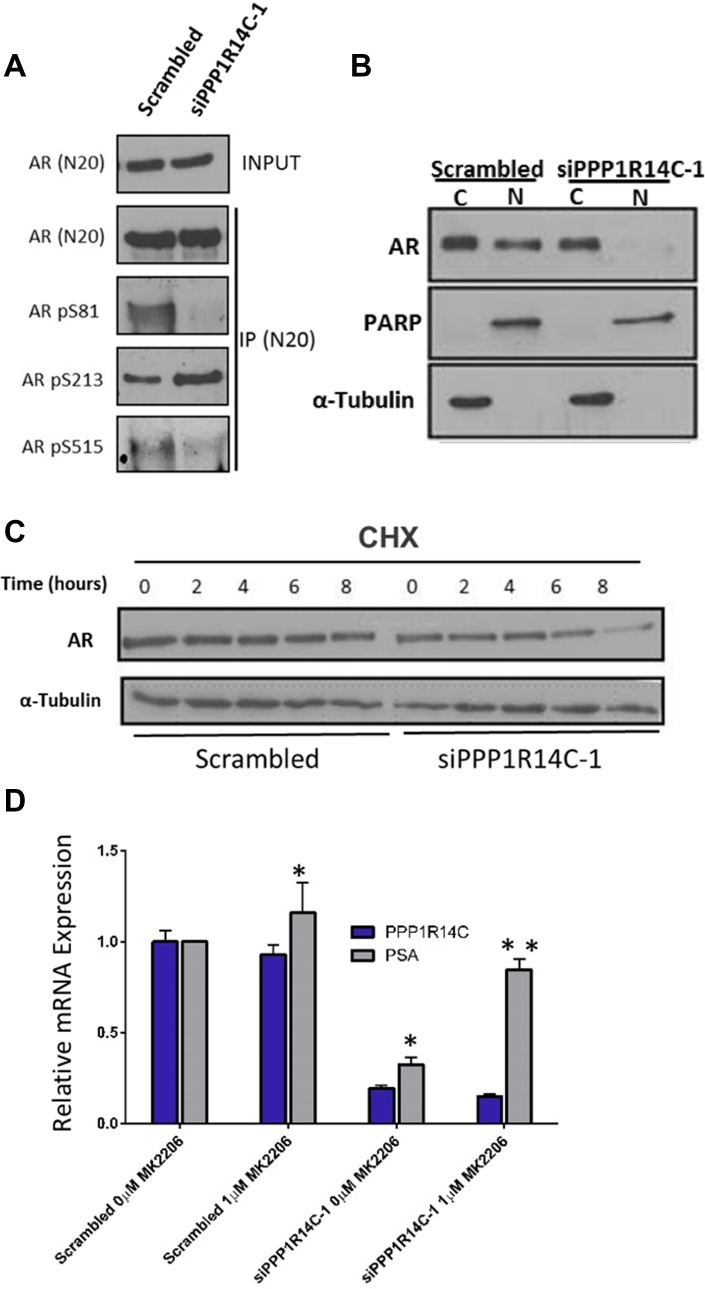
PPP1R14C RNAi depletion prevents cell cycle associated AR phosphorylation, reducing the proliferative and migratory capacity of prostate cancer cells (**A**) Western blot analysis of AR phosphorylation status at S81, S213 and S515 following AR immunoprecipitation in LNCaP cells depleted of PPP1R14C. (**B**) Nuclear-cytoplasmic localization as determined by western blot following nuclear-cytoplasmic fractionation of LNCaP cells depleted of PPP1R14C. (**C**) Western blot analysis of AR protein expression following PPP1R14C RNAi depletion in LNCaP cells cultured in full media and subsequently incubated with 20 µg/ml cycolheximide. (**D**) Impact of AKT inhibition (1 µM MK2206) on *PSA* mRNA expression in LNCaP cells cultured in full media and depleted of PPP1R14C. Data represents 3 independent experiments ± SEM. *P*- values were determined by unpaired student *t* test (^**^, ^*^ denote *P*-values of < 0.001 and < 0.01 respectively).

To gain a better understanding of how PPP1R14C depletion impacts on the molecular functionality of the AR, we interrogated the phosphorylation status of the AR in LNCaP cells depleted of PPP1R14C (Figure 4A). Crucially, a dramatic reduction in AR S81 phosphorylation is observed in PPP1R14C knockdown cells compared to the scrambled control. This phospho-residue has been extensively studied in relation to AR activity [42, 43], and has been shown to correlate with increased nuclear localization and enhanced transcriptional activity. Similarly, a reduction in the phosphorylation of S515 is observed following PPP1R14C depletion. Whilst this site lies within a known MAPK consensus sequence [44], more recent investigations have demonstrated a correlation between increased phosphorylation and CDK1 activity [45], with phosphorylation being associated with enhanced AR activity and disease progression, particularly in the absence of androgen. To confirm the functional impact of the reduced AR phosphorylation at these sites we performed nuclear/cytoplasmic fractionation of LNCaP cells depleted of PPP1R14C following androgen stimulation to investigate the subcellular localization of the AR. Indeed, as depicted in Figure 4B, almost complete ablation of nuclear AR is observed in the absence of PPP1R14C compared to the scrambled control. Finally, a site associated with repression of AR activity [43, 46–48], S213, was found to be significantly enhanced. Phosphorylation of this residue is mediated by AKT and PIM-1, which leads to the subsequent recruitment and ubiquitylation of the AR by MDM2, culminating in AR proteasomal degradation. Further to our previous findings of reduced steady state AR protein levels, knockdown of PPP1R14C in the presence of cycloheximide (CHX) results in accelerated proteasomal degradation compared to the scrambled control (Figure 4C), but more specifically, AR transcriptional activity can be partially rescued following PPP1R14C depletion via exposure to both the proteasomal inhibitor MG132 (Supplementary Figure 4) and the AKT inhibitor MK2206 (Figure 4D).

### RNA sequencing following PPP1R14C RNAi knockdown reveals activation of clinically relevant tumour suppressors and the partial restoration of a non-malignant genotype

**Figure 5:**
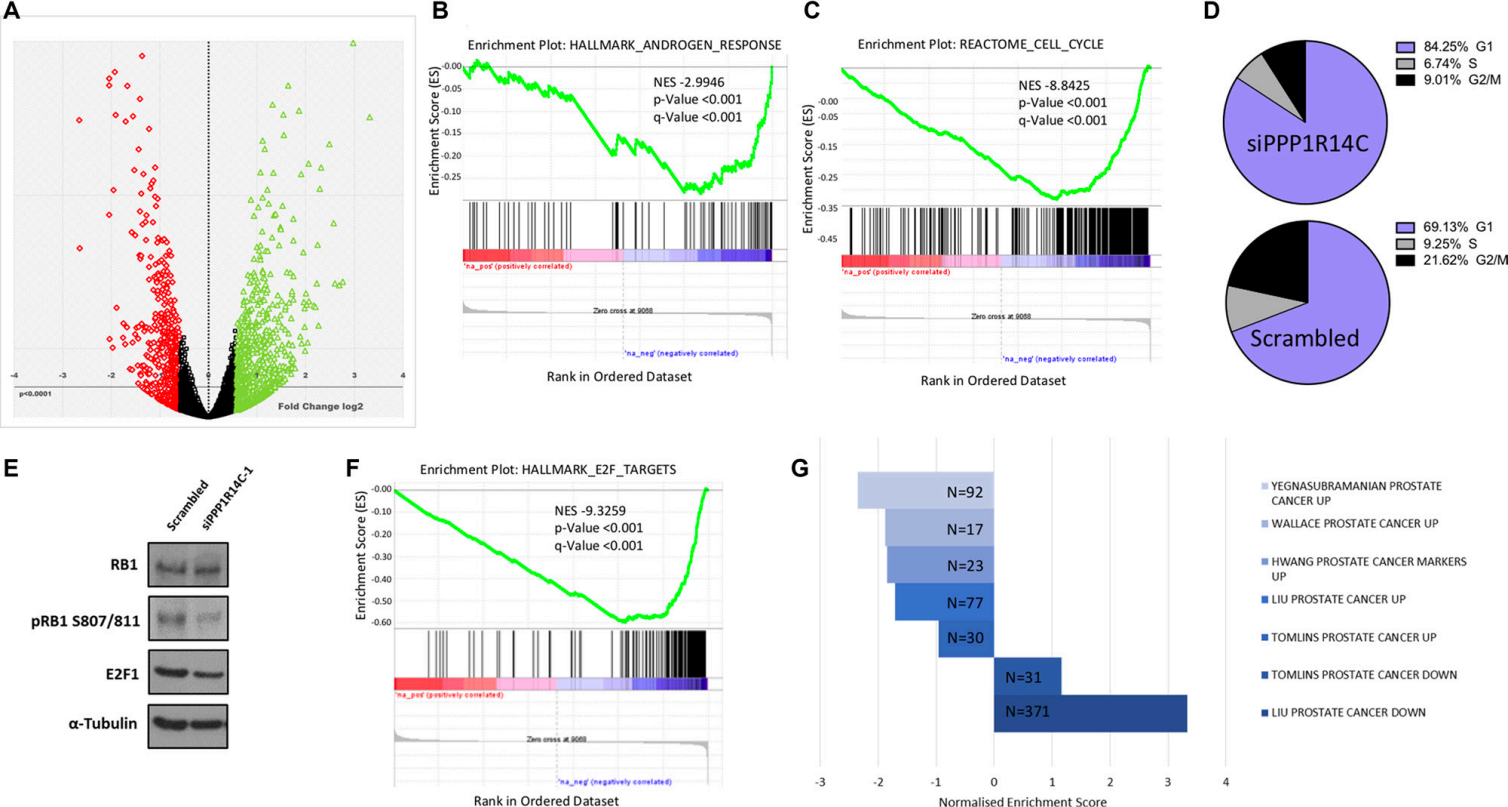
PPP1R14C RNAi depletion partially restores a non-malignant prostate genotype (**A**) Volcano plot representing the differential gene expression quantified by RNA-seq following RNAi depletion of PPP1R14C in LNCaP cells cultured in steroid depleted media supplemented with 10 nM DHT. (**B–C**) GSEA of the PPP1R14C RNAi gene expression profile demonstrates significant and negative enrichment of genes associated with the Hallmark_Androgen_Response geneset(curated geneset derived from 8 independent studies investigating androgen responsive genes) and the Reactome_Cell_Cycle gene set, respectively. (**D**) Representative example of cell cycle analysis of LNCaP cells depleted of PPP1R14C as quantified by PI flow cytometry. (**E**) Western blot analysis of total RB1, pRB1 S807/811 and E2F1 protein expression following PPP1R14C depletion in LNCaP cells cultured in full media. (**F**) GSEA of the PPP1R14C RNAi gene expression profile demonstrates significant and negative enrichment of genes associated with the Hallmark_E2F_Targets geneset (curated geneset derived from 6 independent studies investigating E2F transcription factor target genes). (**G**) GSEA of the PPP1R14C RNAi gene expression profile with 5 independent studies investigating differential gene expression of PC samples vs matched paired normal prostate tissue. Figure displays geneset size and normalised enrichment score.

To gain a greater insight into the role of MLCP on AR signalling we obtained a global gene expression signature by performing RNA sequencing following PPP1R14C RNAi knockdown in the LNCaP cell line in the presence of 10 nM DHT (Figure 5A). Data analysis and subsequent gene set enrichment analysis (GSEA, MSigDB, Broad Institute [49]) revealed that 826 genes were differentially expressed ± 2-fold compared to the scrambled control, including significant repression of the *hallmark androgen response* pathway (Figure 5B, *n* = 101, NES -2.9946, *p* < 0.001). Furthermore, no evidence arose to suggest alteration of additional nuclear hormone receptor activity, e.g. Glucocorticoid receptor. Interestingly, genes involved in cell cycle (Figure 5C, *n* = 421, NES -8–8425, *p* < 0.001), in particular G1-S transition (Supplementary Figure 5, *n* = 112, NES -6.0567, *p* < 0.001), were negatively enriched following PPP1R14C depletion. Confirmation of this impact by FACS analysis revealed that PPP1R14C depletion in LNCaP cells leads to significant G1 cell cycle arrest compared to the scrambled control (Figure 5D, mean ± sem, 69 ± 1.22% vs. 84 ± 0.46%, *p*-value < 0.05).

As previously established, depletion of PPP1R14C is capable of enhancing the activity of MLCP towards its substrates. Consistent with recent reports that MLCP is capable of dephosphorylating RB1 at the inhibitory phospho-residues serine 807 and serine 811 [20, 21], we show a reduction in RB1 phosphorylation at residues S809/S811 by immunoblot following PPP1R14C depletion (Figure 5E). As expected, hypophosphorylation of RB1 at S807/811 results in significant repression in the expression of E2F1 target genes (Figure 5F, *n* = 200, NES -9.3259, *p* < 0.001), providing mechanistic evidence to support the G1 cell cycle arrest observed in Figure 5D. Most importantly, however, genes identified as being significantly up-regulated in PC tissue vs. matched normal prostate tissue in 5 independent genomic studies [34, 50–53], were found to be negatively enriched in LNCaP cells depleted of PPP1R14C compared to LNCaP cells treated with the scrambled control. Conversely, genes found to be significantly down-regulated in PC tissue vs matched normal prostate tissue were found to be positively enriched upon knockdown of PPP1R14C vs Scrambled siRNA (Figure 5G). This would suggest that it may be possible to partially restore a non-malignant transcriptome through enhanced myosin phosphatase activity, and more specifically, through downregulation of PPP1R14C.

### Enhancing myosin phosphatase activity is a viable therapeutic approach in distinct models of PC disease progression

**Figure 6:**
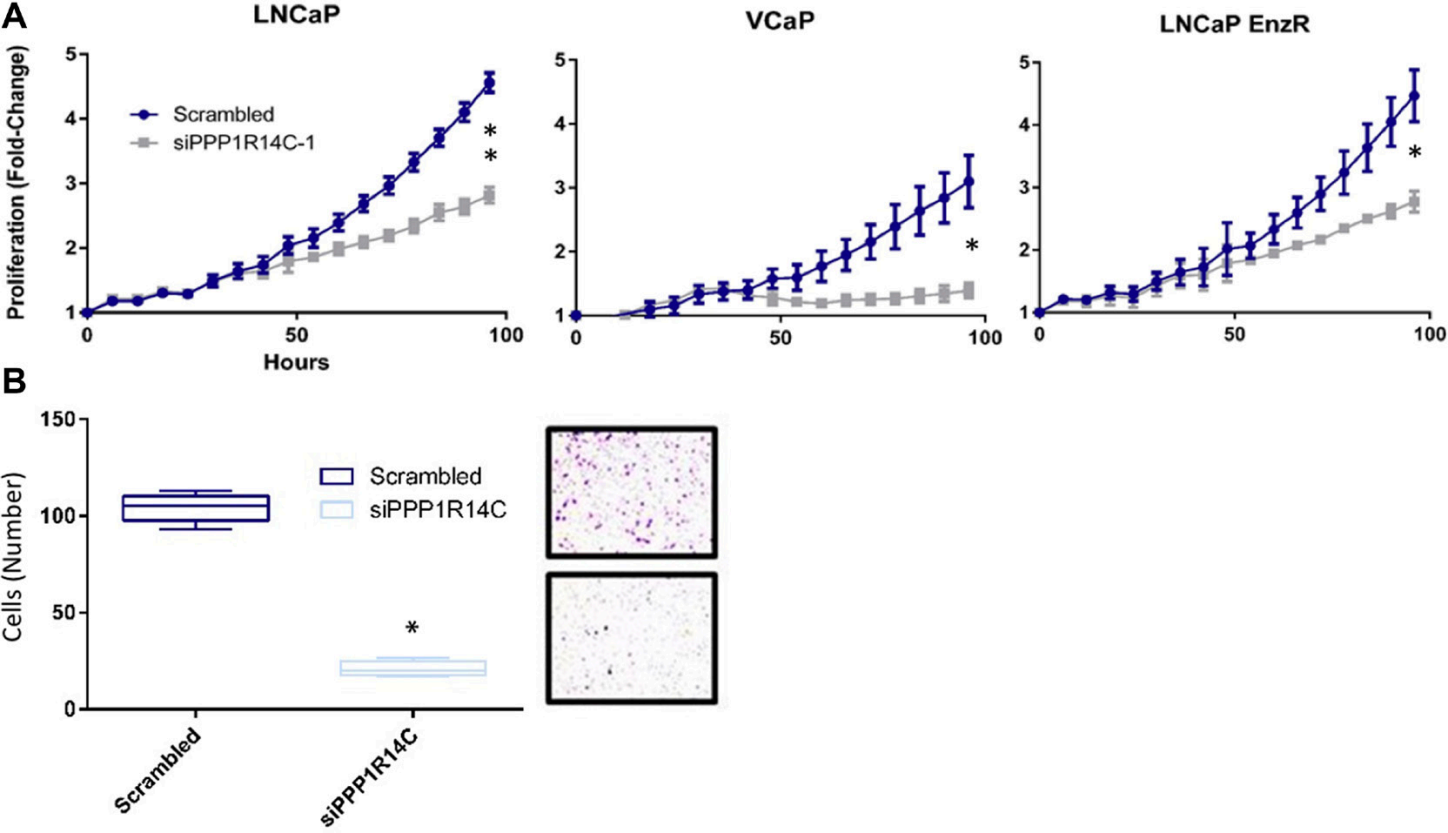
PPP1R14C RNAi depletion significantly impairs prostate cancer cell growth and migration (**A**) Impact of PPP1R14C RNAi depletion on LNCaP, VCaP and LNCaP-EnzR cell growth as quantified by live cell imaging (IncucyteZoom, Essen Bioscience). Cell confluence was measured as a percentage every 6 hours over a 96 hour period with subsequent data represented as fold-change from the scrambled RNAi control. Data represents 3 independent experiments ± SEM. *P*-values were determined by TWO-way ANOVA using Turkey’s comparison test (^**^, ^*^ represents < 0.001 and < 0.01 respectively). (**B**) Impact of PPP1R14C RNAi depletion on LNCaP cell migration was measured using boyden chamber assays and compared to the scrambled RNAi control. Data represents 3 independent experiments ± SEM. ONE-way ANOVA statistical analysis was performed to test statistical significance (^*^ represents a *P*-value < 0.0001).

As our previous studies have identified significant repression of AR transcriptional activity and G1 cell cycle arrest, we sought to further characterise the phenotypic impact of enhanced MLCP activity in distinct PC cell line models. To address this we measured cell growth, using live cell imaging, following manipulation of PPP1R14C expression (Figure 6A). Consistent with a role for PPP1R14C as regulator of AR transcriptional activity and G1-S transition, depletion of PPP1R14C in LNCaP cells results in significant repression of cell growth. Similarly, knockdown of PPP1R14C in VCaP cells in the presence of enzalutamide results in a pronounced reduction in cell growth. Finally, knockdown of PPP1R14C in enzalutamide-resistant LNCaP cells results in significant impairment of cell growth, further confirming the regulatory mechanism imposed by the MLCP-PPP1R14C axis on AR is ligand independent, and as such exploitable irrespective of anti-androgen resistance. Furthermore, the clinical expression of MLCP components could suggest diminished MLCP activity may play a role in tumour metastasis, and as such we sought to investigate the role of PPP1R14C in cell migration. Employment of the boyden chamber assay went on to prove that PPP1R14C depletion results in significant repression of LNCaP cell migration compared to the scrambled control (Figure 6B), providing compelling evidence that PPP1R14C represents a viable therapeutic target in the treatment of both PC and CRPC.

## DISCUSSION

Recent evidence has highlighted the continued benefit of targeting the AR in the treatment of PC under castrate resistant settings [54]. However, it is crucial we develop a greater understanding of the molecular mechanisms underlying the AR signalling axis, particularly in the absence of androgens, to develop novel therapeutic strategies for treatment-resistant patients. It is also becoming increasingly apparent that advanced PC patients harbour complex and heterogenous aberrations of AR co-activators and repressors [55], contributing to AR signalling and treatment resistance. PP1a is one such co-regulator linked to increased AR stability, localization and transcriptional activity, with more recent literature demonstrating continued AR co-activation in castrate resistant settings and activation of AR splice variants. However, here we identify a contrasting role with the activity of the PP1β catalytic subunit in AR regulation, and furthermore, demonstrate for the first time that through association with its regulatory subunits, PP1 imposes both activation and repression of the AR signalling cascade of varying magnitudes in a PP1 holoenzyme specific manner. In addition, we have identified two subunits from the same PP1β holoenzyme complex, MLCP, capable of reciprocally modulating AR function in a dynamic ligand-independent manner. This raises the possibility of modulating the interaction between PP1 and its regulatory subunits as a means of therapeutically targeting PP1 activity in a selective manner. Indeed, disruption of specific PP1 holoenzymes is an approach recently explored by Tsaytler et al to restore proteostasis in a number of human conditions [56, 57].

**Figure 7:**
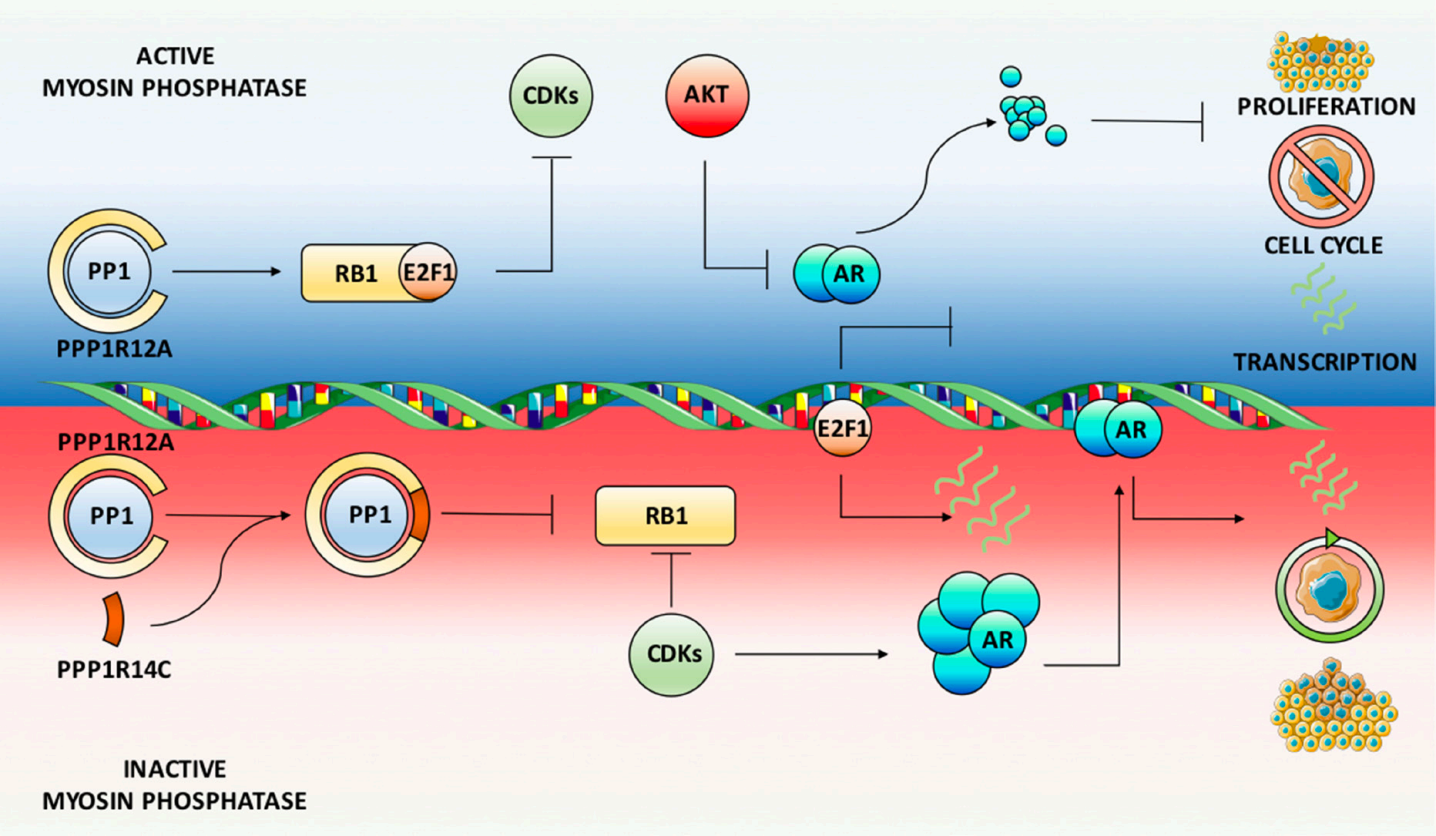
Myosin phosphatase is a dynamic ligand-independent regulator of AR function Schematic summary of MLCP activity on the AR signaling axis. Depletion of the MLCP inhibitory subunit PPP1R14C results in sustained MLCP activity towards its substrates. Amongst these, RB1 is maintained in a hypophosphorylated state, which represses E2F1 mediated cell cycle progression and the associated AR phosphorylation events by CDKs. This leads to enhanced AKT mediated proteasomal degradation of the AR culminating in significant repression of AR target gene expression. Phenotypically this reduces cell cycle progression, proliferation and migration. Conversely, depletion of the MLCP substrate specifying subunit PPP1R12A, representative of MLCP inhibition, results in enhanced AR gene and protein expression and subsequent target gene expression.

Our key findings, schematically summarised in Figure 7, demonstrate that MLCP is a dynamic regulator of AR function in both androgenic and castrate settings, as well as in PC cell lines resistant to current anti-androgens. Crucially, we show that inhibition of PPP1R12A, the substrate specifying subunit for MLCP, significantly increases AR mRNA and protein expression in the absence of androgen, resulting in enhanced AR regulated gene expression. Therefore, repression of MLCP activity under castrate conditions provides a viable route for PC cells to overcome diminished intratumoral androgen concentrations or direct AR antagonism. Indeed, we have identified increased expression of MLCP inhibitory subunits, in combination with reduced PPP1R12A expression in clinical datasets. Furthermore, downstream substrates of MLCP, such as RB1 and PLK1 [58, 59], have been implicated in PC disease progression and treatment resistance, and as such have been identified as significant prognostic markers. Here we show that MLCP modulation provides a plausible route of repressing AR function, whilst enhancing dephosphorylation of crucial downstream molecules including RB1, and as such provides a novel therapeutic approach for targeting AR signalling and aberrant cell cycle machinery in PC and CRPC.

## MATERIALS AND METHODS

### Contact for reagent and resource sharing

Further information and requests for resources and reagents should be directed to and will be fulfilled by the Lead Contact, James Grey (james.grey2@ncl.ac.uk).

### Method details

#### Cell culture

All cells were maintained in RPMI-1640 (Sigma Aldrich) supplemented with 10% (v/v) foetal bovine serum (Sigma Aldrich) and 2 mM L-glutamine (Sigma Aldrich) at 37°C. For steroid-depleted conditions, cells were grown in RPMI-1640 supplemented with 10% (v/v) dextran-coated charcoal-stripped foetal bovine serum (HyClone) and 2 mM L-glutamine. LNCaP-EnzR cells were maintained in the presence of 10 mM enzalutamide.

#### Human phosphatase RNAi screen

Using an automated robot (EpMotion 5070, Eppendorf), 3 independent RNAi oligos targeting 291 phosphatases and 15 control targets were aliquoted into 96 well plates with a final RNAi concentration of 25 nM and stored at –80°C. Prior to the reverse transfection of cells, RNA iMAX (Invitrogen) was incubated with the RNAi for 30 minutes. Then, 5000 LNCaP-PSALuc in DCC media were added to the plates and cultured for 24 hours prior to the addition of 1 mM DHT. Following 48 hours DHT stimulation, 25 µl luciferin reagent (Steady-Glo, Promega) was added to each well prior to luminescence quantification using a FLUOstar Omega plate reader (BMG Labtech). RNAi depletion of each phosphatase target was performed in triplicate, with subsequent target identification validated by qPCR analysis.

#### RNAi transfection, RNA extraction and qPCR

Transient RNAi transfections were carried out using RNA iMAX (Invitrogen) according to manufacturer’s protocol for a total of 72 hours. RNA was then extracted as previously described [8], quantified using a Nanodrop (ThermoFisher) and reverse transcribed using MMLV Reverse Transcriptase (Promega). Target gene expression was evaluated by qPCR using the synthesised cDNA, required primers, SYBR Green (Promega) on an Applied Biosystems 7900HT system.

#### SDS-PAGE and western blotting

Cell lysis and western blot analysis was performed as previously described [7].

#### Cell proliferation, migration and flow cytometry analysis

Cell confluency was measured using the Incucyte Zoom live cell imager (Essen Bioscience) as a surrogate for cell growth over a 96-hour period. Images were collected from various fields every 4 hours, with subsequent confluency data normalised to 0-hour time-point and presented as fold-change. Boyden chamber assays were employed to investigate cell migration as previously described [60] following RNAi depletion of PPP1R14C for 48 hours. Positively migrated cells were fixed, stained, and quantified by eye. Cells within 12 fields of view were quantified for each technical repeat within each experimental repeat (*n* = 3). Flow cytometry was employed to investigate the cell cycle profile of LNCaP cells. Cells were depleted of PPP1R14C for 72 hours prior to preparation and staining with propridium iodide as previously described [61]. Propridium iodide staining was detected using a FACSCalibur (BD Biosciences). Data is mean % of cells from 3 technical repeats, representative of one experimental repeat from a collection of 3 independent experiments.

#### RNA sequencing

LNCaP cells were subject to PPP1R14C RNAi depletion as previously described for a total of 72 hours, including a 24 hour 10 nM DHT stimulation following a 48-hour incubation in steroid depleted conditions. RNA was extracted with RNeasy Plus Kit extraction columns (Qiagen) and quantified with a Nanodrop (ThermoFisher). RNA integrity was calculated through the use of a 2100 Bioanalyzer (Agilent Technologies). Only samples possessing a RIN value > 9 were taken forwarded for [62] sequencing (Non-silencing and siPPP1R14C, *n* = 3). RNA samples were prepared using Stranded Total RNA Sample Prep Kit (Illumina), depleted of ribosomal RNA using the Ribozero Gold Kit (Illumina) and sequenced with 100 bp paired end reads (∼80 million reads/sample) on an Illumina HiSeq 2500 platform. Reads were mapped to human genome hg19 using STAR [63]. Raw read counts were calculated using HTseq [64] prior to differential gene expression analysis with DEseq2. Gene expression analysis was peformed using the gene set enrichment analysis software (Broad Institute) and publicly available datasets from the molecular signatures database (MSigDB, Broad Institute).

### Quantification and statistical analysis

Statistical analysis was performed using GraphPad Prism V7.0. Unless otherwise specified, data are presented as mean ± SEM. Comparisons were performed with the stated statistical tests whose values are represented in the figure legends.

### Data and software availability

siPPP1R14C RNAseq data to be deposited in GEO. All key resources including qPCR sequences, antibodies, RNAi sequences and small molecule reagents can be found in the Supplementary Table 1.

## SUPPLEMENTARY MATERIALS FIGURES AND TABLE





## Abbreviations

(ADT): Androgen Deprivation Therapy
(AR): Androgen Receptor
(CHX): Cycloheximide
(CRPC): Castrate Resistant Prostate Cancer
(DHT): Dihydrotestosterone
(MLC): Myosin Regulatory Light Chain
(MLCP): Myosin Phosphatase
(PC): Prostate Cancer
(PP1): Protein Phosphatase 1
