# Supplementary material for: Differential regulation of the androgen receptor by protein phosphatase regulatory subunits
Source: Oncotarget. 2017 Dec 4;9(3):3922–35. doi: 10.18632/oncotarget.22883 (PMC5790511; doi:10.18632/oncotarget.22883)
Supplement: Supplementary file 1 [file oncotarget-09-3922-s001.pdf]

Differential regulation of the androgen receptor by protein phosphatase regulatory subunits

SUPPLEMENTARY MATERIALS

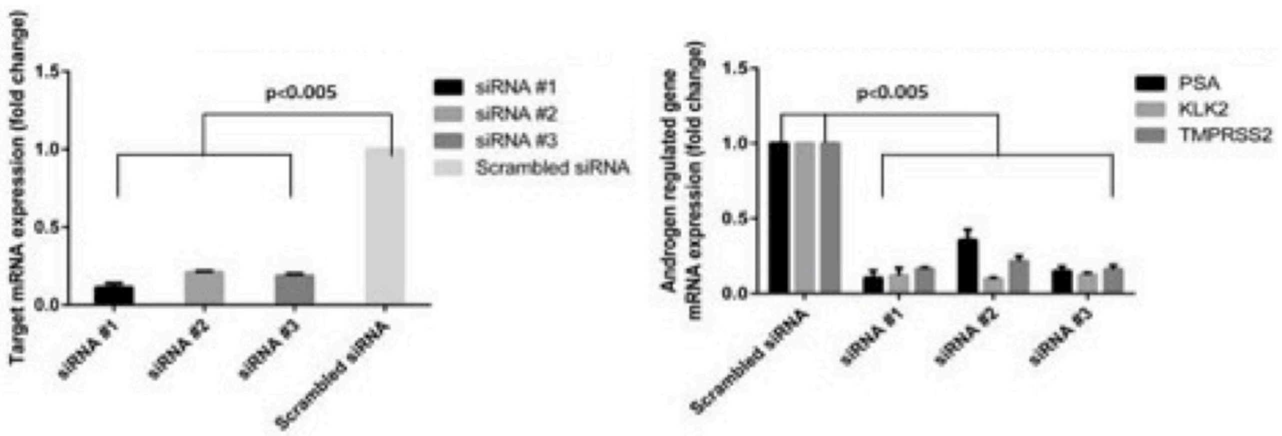

Supplementary Figure 1: PPP1R14C RNAi depletion with 3 independent RNAi oligos results in significant reduction of *PPPIR14C* and AR target gene expression.

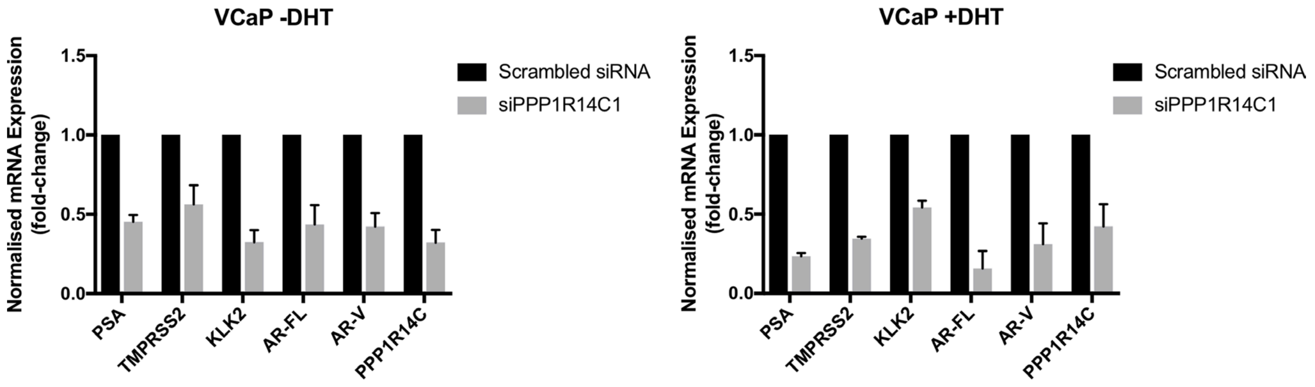

Supplementary Figure 2: PPPIR14C RNAi depletion reduces AR activity in both the presence and absence of androgens in VCaP cells.

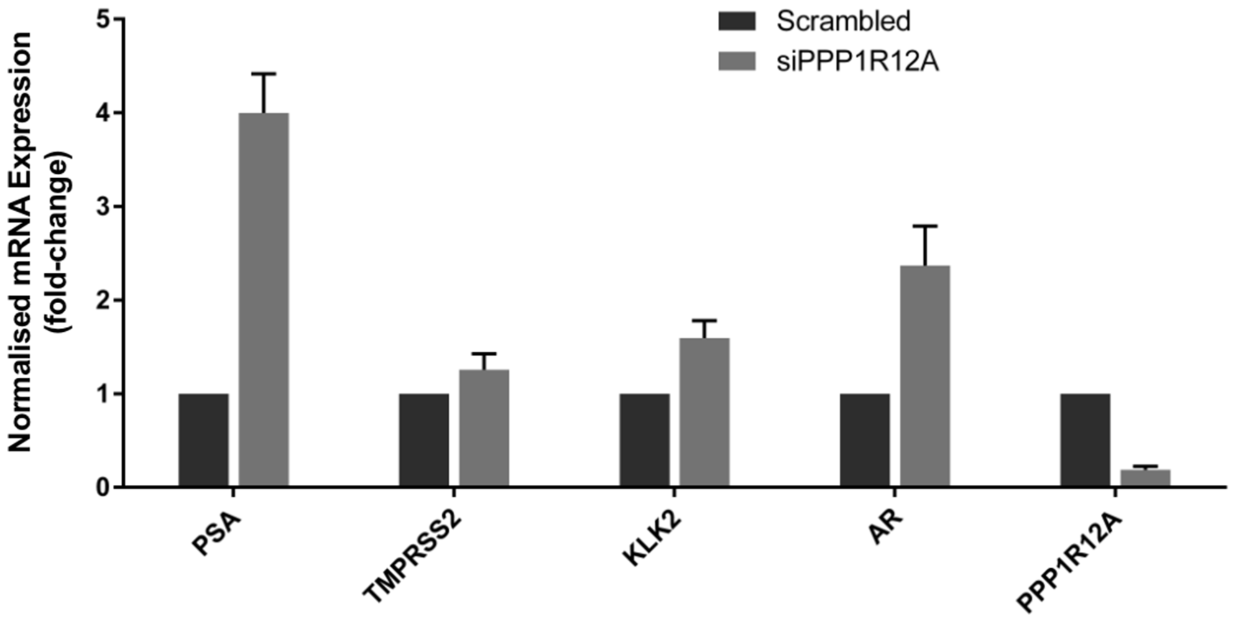

Supplementary Figure 3: PPPIR12A RNAi depletion increases *AR* and AR target gene expression in LNCaP cells in the presence of androgen.

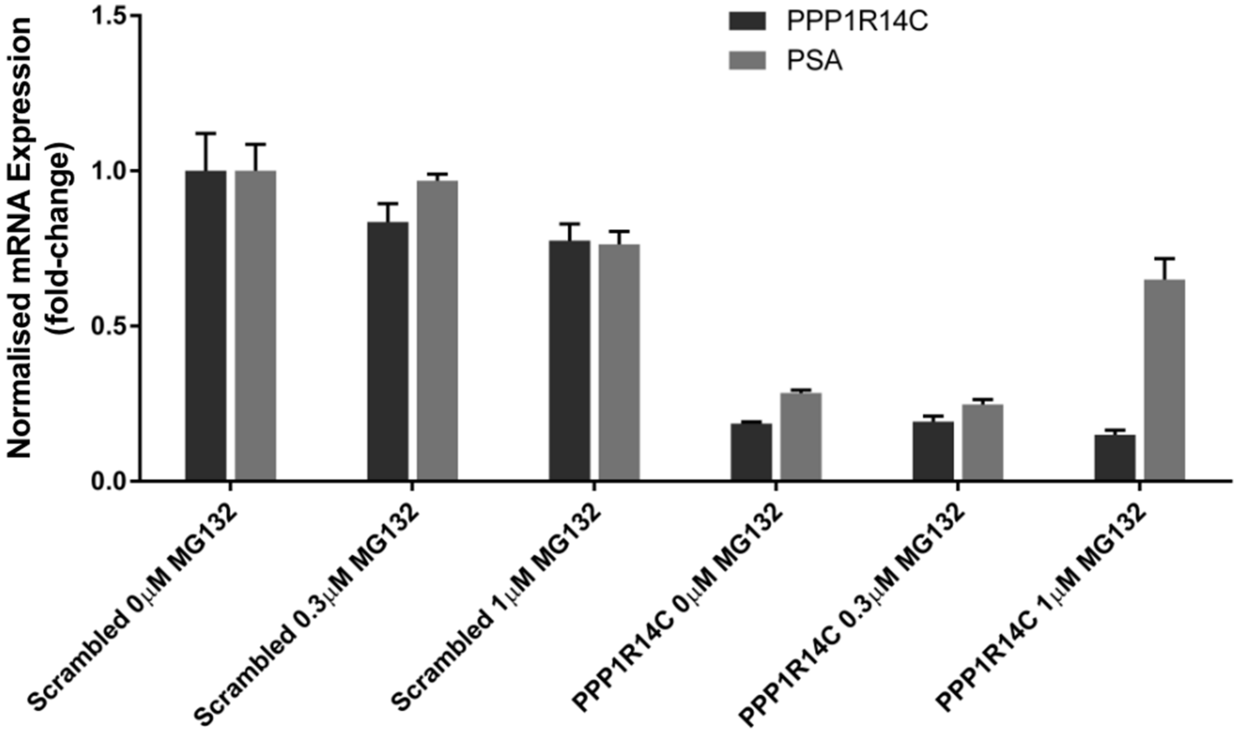

Supplementary Figure 4: PPPIR14C RNAi depletion in the presence of the proteasomal inhibitor MG132 rescues AR transcriptional activity.

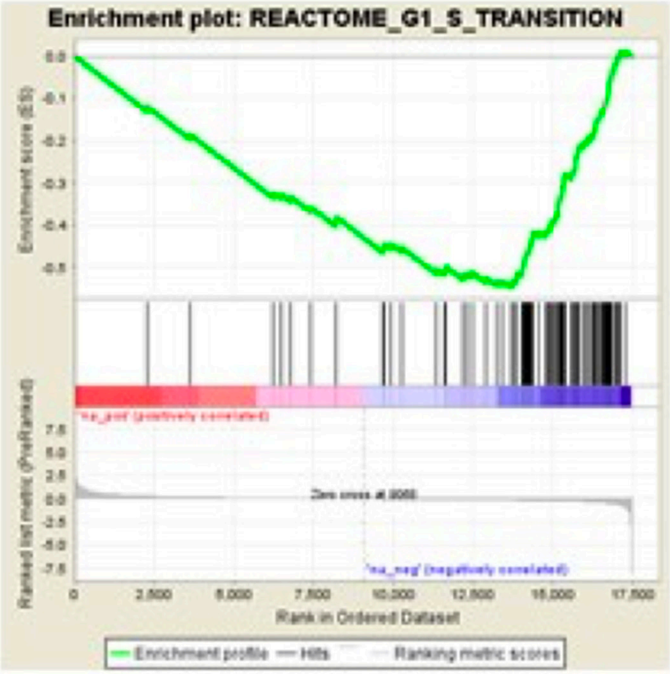

Gene Set	REACTOME G1 S TRANSITION
Enrichment Score (ES)	-0.5478876
Normalized Enrichment Score (NES)	-6.056714
Nominal p-value	<0.001
FDR q-value	<0.001

Supplementary Figure 5: PPP1R14C RNAi depletion results in significant reduction in the expression of genes associated with G1-S transition.

Supplementary Table 1: Key resources table. See Supplementary_Table_1
