# Supplementary material for: Differential regulation of the androgen receptor by protein phosphatase regulatory subunits
Source: Oncotarget. 2017 Dec 4;9(3):3922–35. doi: 10.18632/oncotarget.22883 (PMC5790511; doi:10.18632/oncotarget.22883)
Supplement: Supplementary file 2 [file oncotarget-09-3922-s002.docx]

| Supplementary Table 1 - Key resources table | | |
| --- | --- | --- |
|  |  |  |
| REAGENT or RESOURCE | SOURCE | IDENTIFIER |
| Antibodies | | |
| Rabbit Polyclonal anti-Androgen Receptor | Santa Cruz | Cat# Sc-816 |
| Rabbit Polyclonal anti-Phosphorylated Androgen Receptor Serine 81 | Merck Millipore | Cat# 01-1375 |
| Rabbit Polyclonal anti-Phosphorylated Androgen Receptor Serine 213 | Abcam | Cat# Ab47562 |
| Rabbit Polyclonal anti-Phosphorylated Androgen Receptor Serine 515 | Gift – Dr Joanne Edwards, University of Glasgow | N/A |
| Sheep Polyclonal anti-PPP1R12A | University of Dundee | Cat# DU34962 |
| Mouse Monoclonal anti-Phosphorylated Myosin Light Chain Serine 19 | Cell Signaling | Cat# 3675 |
| Rabbit Polyclonal anti-PARP-1 | Santa Cruz | Cat# Sc1647 |
| Mouse Monoclonal anti-αTubulin | Sigma Aldrich | Cat# T9026 |
| Rabbit Polyclonal anti-Phosphorylated RB1 Serine 807/811 | Cell Signaling | Cat# 9308 |
| Mouse Monoclonal anti-RB1 | BD Pharmingen | Cat# 554140 |
| Mouse Monoclonal anti-E2F1 | Santa Cruz | Cat# Sc251 |
| Swine anti-Rabbit-HRP | Dako | Cat# P0217 |
| Rabbit anti-Mouse-HRP | Dako | Cat# P0260 |
| Rabbit anti-Sheep-HRP | ThermoFisher | Cat# 31480 |
| Goat anti-Rabbit-Alexa 488 | Abcam | Cat# Ab150077 |
| Goat anti-Mouse-Alexa 594 | Abcam | Cat# Ab150116 |
| Chemicals, Peptides, and Recombinant Proteins | | |
| 5αDihydrotestosterone | Sigma Aldrich | Cat# D073 |
| Enzalutamide | Selleckchem | Cat# S1250 |
| Cycloheximide | Selleckchem | Cat# C104450 |
| MG-132 | Sigma Aldrich | Cat# M8699 |
| MK-2206 | Selleckchem | Cat# S1078 |
| Deposited Data | | |
| siPPP1R14C RNAseq Data | This Paper | To Be Deposited |
| Experimental Models: Cell Lines | | |
| LNCaP | ATCC | Cat# CRL-1740 |
| VCaP | ATCC | Cat# CRL-2876 |
| LNCaP-EnzR | This Paper | In-House |
| LNCaP-PSALuc | Gift – Prof Jan Trapman, Erasmus MC, Rotterdam | N/A |
| Oligonucleotides | | |
| Human Phosphatome RNAi Screen | Sigma Aldrich | Cat# S103200 |
| RNAi PPP1R14C #1 Sense ‘GAUAUCAUGACUCUAGCCATT’ | Sigma Aldrich | N/A |
| RNAi PPP1R14C #2 Sense ‘CAAAGGAGGUGGACACUCATT’ | Sigma Aldrich | N/A |
| RNAi PPP1R14C #3 Sense ‘CAGCCUAACCAAGGAUUAUTT’ | Sigma Aldrich | N/A |
| RNAi PPP1R12A #1 Sense ‘AGUACUCAACCAUAAUUAATT’ | Sigma Aldrich | N/A |
| Non-Targeting RNAi Sense ‘UUCUCCGAACGUGUCACGUTT’ | Sigma Aldrich | N/A |
| qPCR Primer PSA | Sigma Aldrich | N/A |
| F: ‘GCAGCATTGAACCAGAGGAG’ |  |  |
| R: ‘AGAACTGGGGAGGCTTGAG’ |  |  |
| qPCR Primer TMPRSS2 | Sigma Aldrich | N/A |
| F: ‘CTGCTGGATTTCCGGGTG’ |  |  |
| R: ‘TTCTGAGGTCTTCCCTTTTCTCCT’ |  |  |
| qPCR Primer KLK2 | Sigma Aldrich | N/A |
| F: ‘AGCATCGAACCAGAGGAGTTCT’ |  |  |
| R: ‘TGGAGGCTCACACACCTGAAGA’ |  |  |
| qPCR Primer PPP1R14C | Sigma Aldrich | N/A |
| F: ‘GGAGAAGAAATGCCAGAGGTAGA’ |  |  |
| R: ‘GCATCAAGAAGATCATCAATGTCAAT’ |  |  |
| qPCR Primer AR | Sigma Aldrich | N/A |
| F: ‘AAGAGAAGTACCTGTGCGCC’ |  |  |
| R: ‘TTCAGATTACCAAGTTTCTTCAG’ |  |  |
| qPCR Primer PPP1R12A | Sigma Aldrich | N/A |
| F: ‘AAGCGCTCCGTCGTCGTCCT’ |  |  |
| R: ‘TCCCCGGAGTAGGCAGAGGT’ |  |  |
| qPCR Primer AR-V7 | Sigma Aldrich | N/A |
| F: ‘AAGAGAAGTACCTGTGCGCC’ |  |  |
| R: ‘TCAGGGTCTGGTCATTTTGA’ |  |  |
| qPCR Primer HPRT1 | Sigma Aldrich | N/A |
| F: ‘TTGCTTTCCTTGGTCAGGCA’ |  |  |
| R: ‘AGCTTGCGACCTTGACCATCT’ |  |  |
